# Process evaluation of a self-compassion-based online group psychotherapy programme for bereavement-related grief: a thematic analysis of the COMPACT feasibility trial

**DOI:** 10.1186/s12904-025-01780-9

**Published:** 2025-05-22

**Authors:** Haruka Tani, Misako Yamada, Shoko Sugao, Yasuhiro Kotera, Yu Uneno

**Affiliations:** 1 Graduate School of Human Sciences, The University of Osaka, 1-2 Yamadaoka, Suita, Osaka Japan; 2 Department of Clinical Psychology, National Center of Neurology and Psychiatry, Tokyo, Japan; 3 School of Health Sciences, University of Nottingham, Nottingham, UK; 4 Department of Palliative Medicine, Kyoto University Hospital, Kyoto, Japan; 5 Center for Infectious Disease Education and Research, The University of Osaka, Osaka, 565-0871 Japan

**Keywords:** Bereavement, Grief, Self-compassion, Online, Internet, Psychotherapy, Thematic analysis, Online psychotherapy, Bereavement care

## Abstract

**Background:**

Bereavement-related grief and prolonged grief disorders are highly prevalent; however, standardised care approaches are lacking. To address this gap, the self-COMPAssion-based online group psyChoTherapy for bereavement-related grief (COMPACT) feasibility trial was developed; it showed promising participant engagement and potential effectiveness. This study aimed to elucidate the mechanisms underlying the impact, contextual factors, and implementation considerations of the COMPACT programme.

**Methods:**

Online semi-structured interviews were conducted and analysed using a deductive reflexive thematic analysis guided by the UK Medical Research Council process evaluation framework. The interviews involved 21 participants and 10 intervention personnel from the COMPACT feasibility trial. The Helpful Aspects of Therapy Form (HATF) was used to guide the interviews, ensuring a focus on identifying mechanisms of impact, contextual factors, and implementation considerations.

**Results:**

Four main “mechanism of impact” themes were identified: common humanity and self-awareness, perceived importance of self-care, deepening self-insights and personal growth, and amplified self-compassion. Two associated “contextual factor” themes (group therapy and a secure programme environment) were highlighted. Additionally, two “implementation consideration” themes (barriers and facilitators) were found, with related contextual themes of group therapy and online delivery.

**Conclusions:**

The core impact mechanisms of the COMPACT programme included accessible online delivery, group work enhancing common humanity, and a safe, supportive environment deepening self-insight, self-care, and self-compassion. Future research should prioritise refining programme content, improving participant assessment, and enhancing training for intervention personnel to enable a randomised controlled trial testing the effectiveness of the intervention.

**Trial registration:**

UMIN000048554, registered 2nd August 2022.

**Supplementary Information:**

The online version contains supplementary material available at 10.1186/s12904-025-01780-9.

## Background

The loss of a loved one often results in profound distress, encompassing psychological, physical, and behavioural reactions [1]. Bereavement-related grief, while a natural response, can significantly increase the risk of prolonged grief disorder (PGD)—characterised by persistent grief that impairs daily functioning for an extended period following bereavement—along with depression, anxiety, and post-traumatic stress symptoms [2, 3]. Despite the widespread occurrence of bereavement-related grief and prolonged grief disorders, there remains a notable absence of standardised care approaches to address these challenges effectively [4].

Individuals with higher levels of self-compassion experience lower levels of grief, indicating that self-compassion-focused therapies are a promising approach for this population [5, 6]. Self-compassion, rooted in Buddhist philosophy, comprises three key elements: kindness towards oneself, recognition of common humanity, and mindfulness of suffering [7]. Gilbert posits that interactions with compassionate caregivers foster a sense of safety [8], which can be particularly beneficial for bereaved individuals. Self-compassion may mitigate self-criticism and guilt, facilitating emotion regulation. This is particularly relevant in the Japanese cultural context, where individuals tend to exhibit a strong self-critical tendency [9], underscoring the need for self-compassion-based interventions.

Online programmes have garnered increasing attention as a means of providing psychological support owing to their ability to mitigate geographical limitations and mental health stigma, while enhancing accessibility and anonymity [10–13]; however, online bereavement support incorporating the concept of self-compassion remains largely unexplored.

Previously, we reported on a feasibility trial of COMPACT, an online group psychotherapy intervention based on self-compassion for bereavement-related grief [14, 15]. COMPACT was designed to mitigate geographical limitations and stigma under the premise that self-compassion can alleviate the psychological burden of grief [11, 13]. Sixty participants were enrolled, with 83.1% (54/60) completing at least four out of five sessions. Secondary outcomes indicated potential improvements in grief, depression, anxiety, and self-compassion, although resilience scores remained unchanged [8]. However, the mechanisms underlying the intervention's effectiveness and its mediators need further investigation.

The UK Medical Research Council(MRC)guidance suggests evaluating complex interventions based on three key themes: mechanism of impact, context, and implementation [16]. Guided by this framework [16], this study aimed to understand the mechanisms through which the intervention impacted grief symptoms, the contextual factors influencing its effectiveness, and the challenges encountered during the implementation of COMPACT. By examining qualitative data that complements the quantitative results of the COMPACT feasibility trial, this process evaluation seeks to address these gaps, contribute to the growing evidence base for self-compassion-based interventions, and provide practical insights for broader implementation in clinical and community settings.

## Methods

This qualitative study included participants and intervention personnel from the COMPACT feasibility trial [8] and received ethical approval from the Kyoto University Graduate School and Faculty of Medicine Ethics Committee (Approved ID: C1565) [14]. All participants provided electronically signed informed consent. The study was registered with the Japanese Clinical Trial Registry (UMIN CTR: UMIN000048554).

### Overview of the COMPACT feasibility trial

The COMPACT single-arm feasibility trial enrolled 60 bereaved adults (≥ 18 years) at least 6 months post-loss (See [15] for details). Psychotherapy, delivered by trained psychologists (intervention personnel), included psychoeducation, self-compassion enhancement, and resilience building (see Additional file 1 for details on intervention content). The 5-week intervention comprised weekly 2-h online sessions, integrating individual and group work, with assignments provided after each session (Table 1). Ten intervention personnel led one to two programmes each, with one psychologist working with 5–6 participants per session alongside an assistant facilitator [8]. Furthermore, the study [8] incorporated quantitative follow-up assessments at 1 and 3 months to evaluate the stability of the intervention's effects.

**Table 1:** Overview of the COMPACT programme timeline

Week 1	• Icebreaking • What is grief for you? • Breathing exercises	Psychoeducation Individual work Group work Post-session assignments
Week 2	• Grief, anxiety, self-care • Mindfulness exercises	
Week 3	• Self-compassion • Three emotion regulation systems	
Week 4	• Image training exercises • Cognitive distortion, ABC model, cognitive reframing	
Week 5	• Loss and gain lines • Compassionate messages from others • Reflections and conclusion	

*ABC* Antecedent, Behaviour, Consequence—a framework for understanding behaviour

### Study subjects

The current study employed qualitative methods to investigate participants and intervention personnel. Participants were recruited from among the COMPACT trial participants who indicated their willingness to be interviewed on a post-session questionnaire. Participants were selected to ensure a balanced sample based on gender and age. Intervention personnel then confirmed the feasibility of interviewing each selected participant, excluding those for whom the interview would be overly burdensome. This convenience sampling approach [17] resulted in a final sample of 21 participants (7 from each cohort). Intervention personnel were recruited via email by HT, targeting all personnel involved in each cohort. The number of participating personnel was 4, 5, and 4 for cohorts 1, 2, and 3, respectively. As four personnel participated in multiple cohorts, the final sample consisted of 10 intervention personnel.

### Procedure

The author YK developed interview guides for participants (Additional file 2) and intervention personnel (Additional file 3) based on the HATF [18]. All interviews were conducted within 56 days of completion of the intervention to enhance participants'recall of the intervention content and their experiences. Participant interviews (45–60 min) involved individual, semi-structured interviews focused on identifying the helpful aspects of the trial. Intervention personnel group interviews (2–2.5 h) were conducted as focus groups to explore their experiences, approaches, perceived effectiveness, and key learning points.

### Analysis

We employed reflexive thematic analysis, which acknowledges the researchers'own perspectives and biases in the interpretation of the data [19]. This analysis was conducted deductively according to the UK MRC’s process evaluation guidance [16]. The approach facilitated a systematic investigation of the intervention's mechanisms of impact, contextual factors, and implementation, allowing us to identify shared understandings related to the intervention. Interviews were audio-recorded, transcribed verbatim by professional transcribers, and anonymised for confidentiality. Initial coding was performed by two researchers, HT and MY (both clinical psychologists). Prior to commencing initial coding, we created a preliminary code list (the initial version of the codebook) based on the research objectives. Initial coding applied codes to relevant segments based on the categories defined by the UK MRC (mechanisms of impact, contextual factors, and implementation). Coding primarily focused on phrases and short utterances, placing emphasis on the subjective emotional experiences of interviewees. HT and MY divided the data for coding and subsequently cross-verified the applied codes to enhance consistency. They evaluated the clarity of code definitions and their suitability to the data, refining the initial version of the codebook by adding, merging, and renaming codes. Following the revision of the initial codebook, a third researcher, SS (a clinical psychologist), joined HT and MY in the collaborative analysis. Themes derived from individual thematic analyses were shared and examined to verify whether the framework-derived themes were supported by the data. The codebook was revised and expanded as the analysis progressed, aiming to capture the data in a manner that preserved participant subjective and emotional experiences.

The final themes were confirmed through consensus discussions among the research team (HT, MY, SS, YK: Psychotherapist, and YU: Internist). This collaborative and iterative analytical approach generated both explicit and latent themes. Focus group interviews followed a similar thematic analysis process.

## Results

**Fig. 1:**
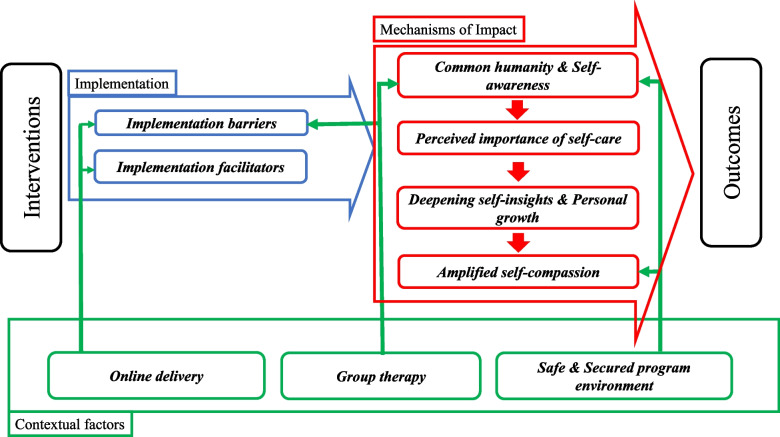
Key mechanisms of impact, contextual factors, and implementation considerations in the COMPACT trial

The mean age of the 21 participants interviewed was 51.6 years (*SD* = 13.8 years), and 85.7% (*n* = 18) were female. Regarding their relationship to the deceased, parents constituted the largest proportion at 47.6% (*n* = 10), followed by children at 33.3% (*n* = 7), and spouses/partners/fiancé(e)s at 14.3% (*n* = 3). The mean time since loss was 12.2 months (median 9 months, range 4–38 months). The 10 professionals interviewed had an average of 16.7 years of experience (median 16 years, range 7–30 years). In terms of workplace, 60.0% (*n* = 6) were affiliated with hospitals and 40.0% (*n* = 4) with universities. Their roles in the COMPACT trial were intervention personnel (70.0%, *n* = 7) and facilitators (30.0%, *n* = 3). Participant characteristics are presented in Table 2. Descriptive quotations, marked with quotation marks, are used to illustrate each theme; for participant statements, ‘P’ refers to intervention personnel, ‘I,’ along with their respective sentence numbers (SN#) (e.g., P-SN: ##). Figure 1 summarises the process evaluation.

**Table 2:** Characteristics of the interviewed patients and intervention personnel

		Interview participants *n* = 21	Interview intervention personnel *n* = 10
Age, mean (SD)		51.6 (13.8)	
Gender (%)	Male	3 (14.3)	
	Female	18 (85.7)	10 (100)
Deceased individual (%)	Parents	10 (47.6)	
	Child	7 (33.3)	
	Spouse/fiancée/partner	3 (14.3)	
	Other	1 (4.8)	
Cause of death (%)	Disease	14(66.7)	
	Suicide	2 (8.0)	
	Other	3 (12.0)	
	Unknown	2 (8.0)	
Time elapsed since bereavement (months)	Mean	12.2	
	Median	9	
	Max	38	
	Min	4	
	Mean		16.7
Professional experience (years)	Median		16
	Max		30
	Min		7
	Hospital		6 (60.0)
Workplace	University		4 (40.0)
	Intervention personnel		7 (70.0)
Role in COMPACT trial	Facilitator		3 (30.0)

This figure illustrates the relationships between the core mechanisms of impact, contextual factors, and implementation considerations identified in the COMPACT trial. Key themes include mechanisms such as common humanity and self-awareness, contextual factors such as group therapy, and barriers and facilitators to implementation. The figure summarises how these elements interrelate to influence the effectiveness of the self-compassion intervention.

### Mechanism of impact (MI)

Analysis revealed four mechanisms of impact themes (Common humanity and awareness, Perceived importance of self-care, Deepening self-insights and personal growth, Amplified self-compassion) and two contextual factor themes (Group therapy, A safe and secure programme environment), encompassing 13 sub-categories and 2,596 meaning units (Table 3).

**Table 3:** Themes and sub-categories of impact mechanisms and associated contextual factors

Themes	Sub-categories
***MI Theme 1*** Common humanity and awareness ***CF Theme 1*** Group therapy ***CF Theme 2*** Safe and secure programme environment	Awareness driven by connection with others Awareness of others (3) Self-awareness of past and present
***MI Theme 2*** Perceived importance of self-care	(1) Acquired knowledge and experiences (2) Helpful practices (3) Awareness through exercises (4) Awareness of the importance of self-care (5) Application the exercise in daily life
***MI Theme 3*** Deepening self-insights and personal growth	(1) Deepening self-insight (2) Sense of change and effectiveness (3) Expectations for the future
***MI Theme 4*** Amplified self-compassion ***CF Theme 2*** Safe and secure programme environment	(1) Learning from self-compassion (2) Experience of safety and security

*Abbreviations: MI* Mechanism of impact, *CF* Contextual factor

#### Mechanisms of impact Theme 1: Common humanity and awareness

Many participants and all intervention personnel expressed empathy for shared experiences, fostering a sense of connection and contributing to healing. Furthermore, all participants recognised a new awareness of their past and present experiences during the COMPACT trial. Descriptive quotations from participants and intervention personnel are provided below to illustrate these points:

*“Being in a group with other people who had lost children, like myself, was healing just by being with people who shared the same grief.” (P-SN: 511)*

*“If I was nodding, and they nodded back at the same time, I felt a sense of reassurance that they were thinking the same way, and I think that was really important.” (I-SN: 95)*

#### Mechanism of impact Theme 2: Perceived importance of self-care

Prior to the intervention, some participants neglected self-care or viewed it as self-indulgent. However, participation in the trial led several to recognise its importance.

*“After facing anxiety and problems, I realised that it is important to care for myself, spend time on self-care, and practice it. I believe that living a healthier life will help me overcome the loss of my loved one.” (P-SN: 4).*

Many participants came to recognise the importance of self-care techniques, such as breathing exercises, by learning and practicing them. Practices they found helpful included breathing exercises (19 participants), reframing (14 participants), meditation (10 participants), and imagery (6 participants). Breathing exercises were particularly popular, with most participants reporting them as beneficial.

*“Reframing was a useful way of thinking for me, as I tend to be self-critical, regardless of grief. Being able to practice it within the programme made me feel like I could do it myself.” (P-SN: 1206).*

Weekly homework assignments in the programme included brief exercises (e.g., breathing exercises and reframing), and participants were encouraged to incorporate these practices into their daily lives. Approximately half of the participants who found the exercises helpful reported continued use.

*“Being able to practice for five weeks and actually test whether it was effective in daily life made a big difference for me.” (P-SN: 184)*

*“Breathing exercises are still easiest for me to do before bed.” (P-SN: 391)*

*“I've been doing breathing exercises at the same time every day since then, so I feel like it's become a habit.” (P-SN: 2125)*

#### Mechanism of impact Theme 3: Deepening self-insights and personal growth

More than half of the participants who practiced the exercises in their daily lives with hope and expectation that the programme would be beneficial became aware of self-compassion and their own cognitive biases, leading to a shift in their thinking.

*“I realised that everyone is responsible for their own life, so my life is mine, and I am responsible for how I think about it. This realisation brought me peace of mind.” (P-SN: 1139).*

Additionally, approximately half of the participants shared that they were able to reflect on their past selves from a broader perspective, which led to deeper self-awareness. Participants expressed feelings of experiencing the effects of the programme's exercises and realising their own growth.

*“I was interested, and well, I feel like I've come to know myself better as I have tried many things.” (P-SN: 2034)*

*“I had many realisations through the programme, and I think those realisations broadened my perspective.” (P-SN: 2898)*

#### Mechanism of impact Theme 4: Amplified self-compassion

Many participants highlighted the significance of self-compassion and confidence gained through interactions within the group and with the intervention personnel.

*“I used to think that being kind to myself was a kind of indulgence and that I needed to have someone do something for me. But I had a new realisation that it is not self-indulgence.” (P-SN: 750).*

Furthermore, most participants spoke about the comfort and safety they experienced during their participation in the programme. This sense of security was identified as a core concept and value of the programme. In particular, many participants specifically mentioned the intervention personnel. They reported feeling reassured by the intervention personnel in the group and experiencing warmth and gentleness from them through their interactions.

*“Just seeing the therapist’s face filled me with a sense of fulfilment and warmth.” (P-SN: 690)*

*“Because clinical psychologists bridged the gap between us, we were able to interact with each other in a safe and comfortable way. I think that was a big factor in my feeling secure enough to participate.” (P-SN: 984).*

Of particular interest, there appeared to be no marked variations in the uptake of self-compassion when comparing individuals bereaved by suicide with those bereaved by other causes.

### Implementation considerations and associated contextual factors

Two implementation considerations themes (1. Implementation barriers and 2. Implementation facilitators) emerged from the analysis, encompassing 15 sub-categories and 1,154 meaning units (Table 4).

**Table 4:** Themes and sub-categories regarding implementation considerations and associated contextual factors

Themes	Sub-categories
***IC Theme 1*** Implementation barriers ***CF Theme 1*** Group therapy ***CF Theme 3*** Online delivery	Challenges in the delivery of individualised care Unique challenges of online delivery Burden faced by intervention personnel Questioning skill mastery Challenges involved in working with individuals with severe grief or trauma Challenges to conduct image training work Unfamiliarity with the exercise content Concerns regarding digital literacy
***IC Theme 2*** Implementation facilitators ***CF Theme 1*** Group therapy ***CF Theme 3*** Online delivery	Optimal accessibility to the programme and programme progress Detailed assessments of participant Adequate intervention personnel training Creative and compassionate care beyond the intervention manual Competent facilitators Expectations of the intervention efficacy Professional development of the intervention personnel

*Abbreviations: IC* Implementation consideration, *CF* Contextual factor

#### Implementation considerations Theme 1: Implementation barrier

Many intervention personnel highlighted programme limitations, including challenges with group sessions, meeting diverse participant needs, and providing individualised care. They reported feeling burdened by a lack of support, particularly the absence of a supervisor. Additionally, they noted the complexities of working with individuals experiencing severe grief or trauma.

*I understand the intervention content intellectually, but the pieces haven’t quite come together yet to form a complete picture. To be honest, we have been experimenting and trying different things as we have implemented the programme. (I-SN: 287)*

While the online format was generally well-received, concerns were raised about accessibility for minority groups.

*For example, I think someone who is deaf would not be able to participate without someone guiding them. (I-SN: 28)*

Some participants offered suggestions for improvement, such as audio guides for meditation and breathing exercises.

*For example, if the facilitators had shared audio guides with images of comfort and safety, participants might have found the homework easier. (I-SN: 3260)*

#### Implementation considerations Theme 2: Implementation facilitators

Participants appreciated the programme's structure, including its 5-week duration, gradual progression, and opportunities for connection through group work. Some participants suggested improvements, such as providing audio guides for meditation and breathing exercises.

*Because it was online, I could participate without worrying about infecting other participants if I got COVID-19. As long as I was feeling well, I could participate. I am glad I was able to participate until the end. (P-SN: 442)*

Some intervention personnel emphasised the need for more detailed assessments of participants, including their grief severity and trauma history. They also highlighted the importance of comprehensive training for intervention personnel, particularly in core concepts like grief and self-compassion. This training, they suggested, would guide programme modifications for future randomised controlled trials.

*I personally feel that the needs might have been quite different depending on the severity of grief and the individual’s personality. (1-SN: 311)*

Most of the intervention personnel demonstrated a commitment to supporting all participants, employing creative approaches beyond the programme manual. They acknowledged the challenges of perceiving non-verbal cues in the online format but remained enthusiastic about the intervention's potential for future research.

*I think it is absolutely crucial to avoid the mindset of simply executing a finished programme. (I-SN: 270)*

They also highlighted the programme's effectiveness and its potential to address the limited bereavement care coverage in Japan. More than half of intervention personnel reported integrating programme elements into their clinical practice, demonstrating its impact on professional development.

*Bereavement care is not widely covered by universal health insurance in Japan, so it is difficult to make progress. I hope this programme can be a first step in that direction. (I-SN: 7)*

### Contextual Factors (CFs)

Recognising that the effectiveness of an intervention may vary across different settings, even when efficacious in a specific context, understanding the contextual factors underpinning COMPACT's impact is crucial [16]. These factors offer insights into potential adjustments needed for the intervention itself. Two contextual themes emerged from the analysis: Group Therapy and a Safe and Secure Programme Environment. The interrelation between these contextual themes and other identified themes is also depicted in Fig. 1.

#### CF Theme 1: Group therapy

This theme demonstrated associations with MI1: Common Humanity and Recognition, and IC Theme 1: Implementation Barriers. Narratives from numerous participants highlighted the pivotal role of the group therapy format in fostering inter-participant connection and enhancing the recognition of common humanity. Conversely, some intervention personnel noted limitations inherent in group therapy, such as the inability to provide individualised support.

*“Also, the environment of being able to do it while feeling that there are others doing it together, not alone, and that it is okay to say that difficult parts are difficult, I think that was significant.” (P-SN: 1636).*

*“When considering that it is a group, not individual, I thought about how to harness group dynamics, group cohesiveness, was important after all."(I-SN: 120)*

#### CF Theme 2: Safe and secure programme environment

This theme appeared to be linked to MI1: Common Humanity and Recognition, and MI Theme 4: Amplified Self-Compassion. A safe and secure programme environment played a crucial role in encouraging self-disclosure and enhancing participants'self-esteem. According to many participants, this safe and secure environment was fostered through connections with the intervention personnel.

*“The fact that we could have conversations with each other with clinical psychologists present was, I think, one of the big factors that made me feel secure enough to participate.” (P-SN: 948).*

*“The staff members have gentle and calm voices, so I felt very secure.” (P-SN: 3217)*

#### CF Theme 3: Online delivery

This theme was associated with IC Theme 1: Implementation Barriers, and IC Theme 2: Implementation Facilitators. Online delivery facilitated access to COMPACT by mitigating geographical constraints and reducing the stigma associated with mental health services. Some participants mentioned being able to participate without concern for infection risk, among other benefits. Meanwhile, some intervention personnel expressed unfamiliarity with online interventions and noted unique challenges specific to the online format.

*“Because it was online and also a group, I honestly feel that it was quite difficult to pay attention to everyone.” (I-SN: 182)*

## Discussion

### Main findings

This study elucidated the mechanism of impact, contextual factors, and implementation considerations of the COMPACT intervention. COMPACT was found to alleviate grief symptoms in participants by fostering recognition of common humanity to reduce loneliness, promoting the practice of self-care to deepen self-insight, and amplifying self-compassion. These mechanisms were reinforced by intervention personnel who provided a safe and secure environment. Implementation challenges were also identified, including interpreting non-verbal cues in the online format and developing strategies to enhance resilience.

### Mechanism of impact in COMPACT

Analysis of interview data using reflexive thematic analysis suggests that the COMPACT process unfolded in a generally stepwise manner. Initially, by encountering fellow participants and intervention personnel and sharing their experiences, participants experienced a reduction in feelings of isolation and gained reassurance that they were not alone in their suffering (Common Humanity and Recognition). Subsequently, through learning and practicing self-care techniques such as breathing exercises and reframing, some participants recognised the importance of self-care and began incorporating it into their daily routines (Recognition of the Importance of Self-Care). Building upon these experiences, a subset of participants appeared to undergo a shift in perspective, deepening their understanding of themselves (Deepening Self-Insight and Personal Growth). Ultimately, through these cumulative experiences, participants seemed to recognise and embrace the significance of self-compassion, fostering a greater sense of kindness towards themselves (Amplification of Self-Compassion). These stages are not strictly demarcated but rather are considered to be mutually influential and interact synergistically to produce the overall effect of COMPACT.

However, it is crucial to acknowledge that this proposed mechanism remains hypothetical. It may not apply uniformly to all individuals, and variations in the extent and manifestation of its effects are to be expected. This hypothesised mechanism, derived from interview data focusing on participants'emotional experiences, necessitates further validation through quantitative data and long-term follow-up studies.

### Interrelationship between participants and intervention personnel

The aforementioned themes illuminate the interrelationship between participants and intervention personnel. COMPACT appears to have been effective in enabling most participants to recognise common humanity, reduce feelings of loneliness, deepen their understanding of self-compassion, promote the practice of self-compassion, and provide an experience that alleviated emotional distress. Intervention personnel emphasised that COMPACT fostered a warm and accepting atmosphere where participants felt safe to engage in self-disclosure. Intervention personnel warmly supported participants as they gained a sense of security and became capable of discovering new solutions. Participants indicated that the compassion (encompassing consideration, empathy, and ingenuity) demonstrated by the intervention personnel enabled them to engage with the programme within a safe and secure environment. Thus, COMPACT can be understood as having a therapeutic effect, wherein participants'self-reflection and the compassion of the intervention personnel who warmly observed and supported this introspection synergistically promoted participants'psychological growth and facilitated their recovery from grief.

### Sense of safety and security and expertise of intervention personnel

The mechanism of impact in the COMPACT trial may have been reinforced by the contextual factor of a sense of safety and security. Participants reported feeling safe and secure, which they attributed to the exceptional creativity and care of intervention personnel that extended beyond the programme manual. This report aligns with Gilbert’s theory that interactions with compassionate caregivers foster a sense of safety [8], reduce vigilance for threat, and encourage exploration. The strong connection between participants and intervention personnel appeared to enable participants to explore their inner selves safely and confidently, which is crucial for personal growth. This study reaffirmed the importance of high-quality interventionist skills for programme effectiveness, a finding consistent with prior research [20].

### Sense of connection with others

Throughout the COMPACT programme, participants encountered others with similar experiences during group therapy, fostering a sense of common humanity and connection—key components of self-compassion. The mechanism of impact revealed that participants'experience of common humanity served as a catalyst for subsequent experiences. Grief-related distress includes feelings of loneliness, anxiety, and hopelessness [21]. Even without malice, casual remarks from others can hurt bereaved individuals and make them feel misunderstood [22]. For participants who often feel isolated and trapped, discovering that others share their pain was likely a profound experience. Discovering that others share their pain offered solace and mitigated potential hurts from casual and unintentional remarks. Furthermore, while previous research has highlighted the role of shared humanity in peer support [23], this study underscores the unique potential of group therapy to cultivate such meaningful connections.

### Practical benefits of self-care

The mechanism of impact suggested that experiencing common humanity and the sustained practice of programme exercises, such as breathing exercises and mindfulness, may have led to self-insight and self-growth. This indicates that while incorporating new practices into daily life can be challenging [24], the flexible homework assignments in the programme, designed to be completed at a convenient time, facilitated the development of routines. The benefits and importance of self-care have been previously described, and our study aligns with previous research [25, 26].

Future implementation should focus on enhancing participants’ self-efficacy, ensuring that exercises are simple and easy to understand, and minimising psychological barriers to practice.

### Identified future challenges

While our previous quantitative report showed no significant improvement in resilience [15], qualitative data suggest a pressing need to prioritise this aspect. Imagery training, intended to enhance resilience, sometimes amplified distress as participants focused on images of the deceased [15], highlighting the potential for triggering painful memories. Additionally, participants encountered challenges in applying learned skills to personal experiences, such as imagining a safe place, which for some was associated with the deceased. These challenges underscore the need to tailor interventions more closely to individual grief experiences. One participant poignantly stated,"Since my husband passed away, there is no safe place left in this world."Further refinement of resilience-enhancing methods is needed. Future research could explore alternative approaches to resilience training that minimise emotional distress, such as adaptive imagery techniques or personalised coping strategies tailored to individual grief experiences.

Additional areas for improvement include participant assessments during recruitment to determine suitability for online psychotherapy, enhanced training for intervention personnel, and the evaluation of the long-term stability of intervention effects. The online format presents challenges in perceiving non-verbal cues, and the group setting can limit individualised attention. The introduction of a supervisor could address these challenges, support intervention personnel, and ultimately enhance programme effectiveness. Future research should consider incorporating qualitative or quantitative follow-up at 3, 6, or 12 months to evaluate the long-term stability of the intervention effects.

Furthermore, when applying the findings of this study to other countries, it is imperative to consider cultural and healthcare system differences. While Japan exhibits a cultural background characterised by a strong tendency toward self-criticism, this may not apply in other cultural contexts. Additionally, although bereavement care is not widely covered by social insurance in Japan, the situation may differ in other nations. Implementing COMPACT in different countries would necessitate adapting the programme to account for these disparities.

### Strengths and limitations

This qualitative study explored the mechanisms and effects of the intervention through participant experiences [27]. Interviews with both participants and intervention personnel provided a multifaceted understanding of the intervention.

However, several limitations warrant consideration. First, while the MRC guidelines and the HATF provided valuable guidance in research design, data collection, and analysis, we also acknowledge the inherent limitations of adhering to pre-existing frameworks. For instance, the MRC process evaluation guidelines may not fully capture elements unique to the COMPACT intervention. The HATF did not allow for in-depth exploration of participants'bereavement experiences. Future research should incorporate inductive approaches and develop more tailored evaluation methods to capture elements specific to COMPACT.

The second limitation concerns the generalizability of the findings. Reliance on participant consent for interview participation may have introduced bias towards a positive perception of the programme. Additionally, while participants presented diverse backgrounds in terms of bereavement experiences, age, and gender, the utilisation of reflexive thematic analysis resulted in the generation of common themes. Consequently, the inability to capture the individuality of bereavement experiences represents a limitation of this study. Future research is encouraged to pursue narratives that explore the experience of intervention, starting from the bereavement period.

The third limitation is the low number of male participants in the COMPACT trial (7 out of 60 participants). This may be influenced by the cultural stigma surrounding men's mental health in Japan. Future research should explore strategies to increase male participation.

## Conclusions

The COMPACT programme's core mechanisms of impact and associated contextual factors included easy access through online delivery, enhancement of a sense of common humanity through group work, and deepening of self-insight, self-care, and self-compassion within a safe and secure programme environment. Addressing issues related to programme implementation is essential for successfully conducting a randomised controlled trial and achieving broader social implementation of the intervention. These findings contribute to the broader evidence base for online psychological interventions, particularly in addressing bereavement-related grief and fostering emotional resilience in diverse populations.

## Supplementary Information

Additional file 1: Details of the COMPACT trial. This document provides a detailed description of the COMPACT intervention to enhance the readers' understanding of its methodology within this study.

Additional file 2: Patient interview guide. This file contains the semi-structured interview guide developed for patients participating in the COMPACT trial. It includes detailed instructions and questions designed to gather insights on their experiences, learnings, and feedback about the trial, based on the HATF.

Additional file 3: Therapist interview guide. This file contains the semi-structured interview guide developed for therapists participating in the COMPACT trial. It includes prompts designed to explore their experiences, challenges, and perspectives on implementing the self-compassion intervention in clinical practice.

## Data Availability

The data generated or analyzed during the current study are not publicly available due to participant privacy protection. However, the data used in this study are available from the corresponding author upon reasonable request.

## Abbreviations

COMPACT: Compassion-focused Online Psychotherapy for bereavement-related grief
UK MRC: United Kingdom Medical Research Council
CF: Contextual Factor
MI: Mechanism of Impact
RCT: Randomised Controlled Trial
UMIN CTR: University Hospital Medical Information Network Clinical Trials Registry
ABC: Antecedent, Behaviour, Consequence
