# Supplementary material for: Process evaluation of a self-compassion-based online group psychotherapy programme for bereavement-related grief: a thematic analysis of the COMPACT feasibility trial
Source: BMC Palliat Care. 2025 May 22;24:144. doi: 10.1186/s12904-025-01780-9 (PMC12101004; doi:10.1186/s12904-025-01780-9)
Supplement: Supplementary file 1 — Additional file 1: Details of the COMPACT trial. This document provides a detailed description of the COMPACT intervention to enhance the readers' understanding of its methodology within this study. [file 12904_2025_1780_MOESM1_ESM.docx]

**Details of the COMPACT trial**

**Purpose:**

This document aims to provide a detailed description of the COMPACT intervention and enhance readers' understanding of its methodology within this research.

**Overview:**

COMPACT is an online group psychotherapy program grounded in self-compassion, designed for individuals experiencing bereavement-related grief. The intervention intensity required to enhance self-compassion is reported to be a total of 11 hours of intervention, including self-learning . Therefore, this program is structured over five weeks, consisting of two-hour online sessions held weekly, with post-session assignments given after each session.

【Reference】 Kotera Y, Van Gordon W. Effects of Self-Compassion Training on Work-Related Well-Being: A Systematic Review. Frontiers in psychology. 2021;12:630798.

**Participants:**

The inclusion criteria for participants were as follows:

・Individuals aged 18 years or older who were at least 6 months post-bereavement.

【Reference】 Jerome H, Smith KV, Shaw EJ, et al. Effectiveness of a Cancer Bereavement Therapeutic Group. Journal of loss & trauma. 2019;23(7):574-587.

・Individuals capable of participating in online sessions and independently undertaking post-session assignments.

・Individuals who possess a smartphone or PC and have a stable internet connection.

**Intervention Personnel:**

The criteria for intervention personnel were as follows:

・Certified Public Psychologist (Japan), clinical psychologist, or individuals holding a master's degree in clinical psychology or equivalent qualifications.

・Individuals deemed by the researchers to possess the qualities necessary to provide care for significant others.

・Individuals who had undergone more than 10 hours of structured training specific to the interventions used in this trial.

**Session Content for Each Week:**

**Week 1: Introduction**

- Establishing Ground Rules: Setting and confirming rules such as practicing empathetic communication, defaulting to muted audio, and using the raise-hand button for speaking.
- Self-Introductions: Brief, fact-based self-introductions without delving into the details of the bereavement.
- Breathing Exercises: Practicing breathing exercises together as a group to foster a sense of unity. Repeating the 4-second inhale, 7-second hold, and 8-second exhale cycle approximately five times.

**Week 2: Psychoeducation on Bereavement Grief**

- Understanding Grief: Explanation that grief is a natural emotional response and can be an overwhelming emotional reaction. Description of the five stages of grief (denial, anger, bargaining, depression, acceptance).
- Understanding Bereavement-Related Anxiety and Recognizing the Importance of Self-Care: Explanation that while grieving, individuals may feel anxious due to a sense of lack of control over their lives. Explanation of the importance of self-care, as neglecting sleep and self-care can exacerbate anxiety.
- Mindfulness Practice: Explanation of mindfulness as paying attention to the "present moment" and experiencing things as they are, without subjective judgment. Practice of mindfulness breathing exercises.

**Week 3: Self-Compassion**

- Understanding Self-Compassion: Explanation of the three components of self-compassion: self-kindness, common humanity, and mindfulness.
- Understanding the Three Emotion Regulation Systems: Explanation of the three emotion regulation systems: threat, drive, and soothing. Emphasis on the importance of activating the soothing system.
- Imagery Work: Practice of imagery work after understanding self-compassion and the three emotion systems. Participants are guided to imagine someone who offers them compassion, envision what that person would say to them about their difficulties, and experience the feeling.

**Week 4: Enhancing Resilience**

- Understanding Cognitive Distortions: Learning about cognitive distortions (biases in thinking patterns). Participants consider what kind of "unwarranted assumptions" they might be holding and examine whether they are distorting reality.
- Reframing: Experiencing how changing the "frame," which includes values, thinking habits, and assumptions, can alter the overall impression and perception of a situation.

**Week 5: Conclusion**

- Loss and Gain Lines: Participants list what they have lost and gained throughout their lives, from birth to the present. They then discuss their feelings about what they have written.
- Messages from Compassionate Others: Participants imagine three people who offer them compassion (e.g., grandmother, friend, mentor). Other participants and intervention personnel take on the roles of these compassionate individuals, delivering messages of care. Participants discuss how they feel receiving these messages of compassion.
