# Supplementary material for: Process evaluation of a self-compassion-based online group psychotherapy programme for bereavement-related grief: a thematic analysis of the COMPACT feasibility trial
Source: BMC Palliat Care. 2025 May 22;24:144. doi: 10.1186/s12904-025-01780-9 (PMC12101004; doi:10.1186/s12904-025-01780-9)
Supplement: Supplementary file 2 — Additional file 2: Patient interview guide. This file contains the semi-structured interview guide developed for patients participating in the COMPACT trial. It includes detailed instructions and questions designed to gather insights on their experiences, learnings, and feedback about the trial, based on the HATF. [file 12904_2025_1780_MOESM2_ESM.docx]

**Additional file 1**

**Appendix A: Interview guide (patient)**

**[Prior to recording]**

Thank you for agreeing to participate in this interview. Before we begin, I would like to go over some important points. Please confirm that you are in a quiet and comfortable location where you can speak freely. Today, we will be discussing your experiences with the COMPACT trial. The information you share will be used solely for research purposes. Please feel encouraged to express your thoughts and feelings openly and honestly, without any concern for my presence or the setting.

This interview will be audio-recorded. To ensure your privacy, all identifiable information, such as your name, will be removed from the recording. Any remaining identifying details will be kept confidential within the audio file until its disposal. Once all interview recordings have been analyzed, the audio files will be deleted. Do you consent to the recording of this interview?

Please feel free to ask any questions or raise any concerns you might have.

**[Begin Recording]**

**1. Experience with the COMPACT Trial**

Please share your honest thoughts and feelings about your experience participating in the COMPACT trial. What aspects of the trial did you find most important or beneficial? This could include anything you said or did or anything the intervention staff said or did.

**2. Importance and Learnings from the Trial**

Please share your thoughts on the significance and usefulness of this program. What did you learn from participating in this program?

**3. Effectiveness and Impact of the Trial**

To what extent did this program help or hinder you? Please rate your response on a scale of 0 to 100, where 0 means "completely hindered" and 100 means "completely helped."

**4. Timing of the Most Helpful/Hindered Event**

During which session of the trial did the most helpful or hindering event occur?

**5. Duration of the Event's Impact**

How long did the impact of that event last?

**6. Other Helpful Aspects**

Were there any other aspects of this session that were especially helpful?

Yes:

(a) Please rate how helpful that aspect was.

(b) Briefly describe what made it helpful.

No:

**7. Obstacles During the Session**

Did you encounter any obstacles during the session?

Yes:

(a) Please rate how hindering that obstacle was.

(b) Briefly describe the obstacle.

No:

**8. Additional Comments**

Do you have any other comments or feedback about the trial?

**[End of Interview]**

Thank you very much for your time today.
