# Supplementary material for: Process evaluation of a self-compassion-based online group psychotherapy programme for bereavement-related grief: a thematic analysis of the COMPACT feasibility trial
Source: BMC Palliat Care. 2025 May 22;24:144. doi: 10.1186/s12904-025-01780-9 (PMC12101004; doi:10.1186/s12904-025-01780-9)
Supplement: Supplementary file 3 — Additional file 3: Therapist interview guide. This file contains the semi-structured interview guide developed for therapists participating in the COMPACT trial. It includes prompts designed to explore their experiences, challenges, and perspectives on implementing the self-compassion intervention in clinical practice. [file 12904_2025_1780_MOESM3_ESM.docx]

**Additional file 2**

**Appendix A: Interview guide (**therapists**)**

**[Prior to recording]**

Thank you for participating in the COMPACT trial and agreeing to this interview. The purpose of this interview is to gain a deeper understanding of therapists' experiences within the COMPACT trial. The information you share will be used solely for research purposes. Please feel comfortable sharing your honest thoughts and feelings.

This interview will be audio-recorded. To ensure your privacy, all identifiable information, such as your name, will be removed from the recording. The audio recording will be securely deleted after the analysis is complete. Do you consent to the recording of this interview? Please let me know if you have any questions or concerns.

**[Begin Recording]**

**1. Experience with the COMPACT Trial**

Please share your honest thoughts and feelings about your experience participating in the COMPACT trial.

**2. Key Considerations in Providing the Intervention**

As a provider of the self-compassion intervention, what were your most important considerations?

**3. Effectiveness and Challenges of the Intervention**

From your perspective as a provider, which aspects of the intervention were most effective for participants? Conversely, which aspects were least effective?

**4. Learnings and Insights from the Intervention**

What learnings and insights did you gain from providing the intervention?

**5. Difficulty of Implementing the Intervention**

Did you find the self-compassion intervention easy or difficult to implement?

**6. Suitability of the Intervention to Needs**

Do you feel that the self-compassion intervention met the needs of the bereaved individuals?

**7. Potential for Implementation in Healthcare Settings**

Do you think healthcare settings in Japan would generally be receptive to implementing this type of intervention? What about your own workplace?

**8. Application to Daily Practice**

How confident are you in your ability to apply the self-compassion techniques you learned in the workshop to your daily practice?

**9. Integration into Daily Practice**

Do you think the self-compassion intervention could be effectively integrated into your daily clinical practice?

**[End of Interview]**

Thank you very much for your time today.
